# The use of saliva steroids (cortisol and DHEA-s) as biomarkers of changing stress levels in people with dementia and their caregivers: A pilot study

**DOI:** 10.1177/00368504211019856

**Published:** 2021-05-24

**Authors:** Tores Theorell, Gabriella Engström, Helena Hallinder, Anna-Karin Lennartsson, Jan Kowalski, Azita Emami

**Affiliations:** 1 Stress Research Institute, Stockholm University, Stockholm, Sweden; 2 Department of International Health, Karolinska Institute, Stockholm, Sweden; 3 Dalarna University School of Health and Welfare, Sweden; 4 Memory Clinic, Stockholm Sjukhem, Stockholm, Sweden; 5 Institute of Stress Medicine, Gothenburg; 6 JK Biostatistics, Karlbergsvägen, Stockholm, Sweden; 7 School of Nursing, University of Washington, Seattle, WA, USA; 8 Division of Occupational Therapy, Department of Neuroscience, Care Sciences & Society (NVS), Karolinska Institutet, Stockholm, Sweden

**Keywords:** Dementia, home care, music, family caregiver, saliva cortisol, DHEA-S, stress

## Abstract

The rationale was to explore the efficacy/sensitivity of using morning and evening cortisol levels as biomarkers for stress reduction in persons with dementia (PWDs) and their family caregivers (FCGs) participating in a music intervention program. Thirty-two PWD and their FGC were recruited to an 8-week, home-based music intervention program. Daily home-based collection of saliva samples took place at bedtime and upon awakening. Cortisol was analyzed in the morning and evening saliva samples and DHEA-s in the morning samples. Trends over 40 workdays (15–40 observations per subject) were assessed using linear regression analysis. Twenty-three PWD (72% of invited, 16 men and 7 women, age 69–93) and 24 caregivers (75%, 8 men and 16 women, age 37–90) completed the intervention for at least 6 weeks and were included in the analysis. One-fourth of the PWD and FCG had decreasing evening cortisol, accompanied by decreasing morning cortisol levels. In one-fourth of the participants the ratio between cortisol and DHEA-S in the morning samples was improved, indicating improved balance between energy mobilization and regeneration. Several participants showed no significant endocrine change. There was a statistically significant (two-sided test) correlation within the PWD-caregiver dyads in evening cortisol trend and a statistically significant decrease (two-sided test) in the morning-evening cortisol slope for the FCG group. Reduction in stress, as measured by evening cortisol, was observed in a substantial number of the participants. Recording endocrine stress is helpful for the unbiased assessment of the intervention.

## Introduction

Dementia is an incurable, progressive neurodegenerative process that results in a loss of mental ability, and eventually death. It afflicts more than 47 million people worldwide and this number is expected to double every 20 years.^1^ The Western European dementia prevalence rate is estimated to be 7.9% of those over the age of 60.^2^

Dementia has a strong negative impact on an even larger group of people – the family caregivers who provide a substantial majority of the care for those with dementia. Family caregivers are exposed to a psychosocially and physically demanding situation. The behavioral unpredictability of a person with dementia leads to high levels of stress and depression in FCGs.^3^

Although FCGs provide the bulk of dementia care, relatively little research has been done on evaluating affordable, accessible, practical non-pharmacological interventions to assist them in managing challenging PWD behaviors and reducing stress levels.

One non-pharmacological intervention that has come to the fore in recent years is music, including singing. Research to date shows that PWDs respond favorably to music in many forms.^4^ Listening to familiar music and caregiver singing during personal care activities reduces PWD resistance to care, increases positive interactions between PWDs and caregivers, and reduces caregiver stress level.^5–7^

However, the means for evaluating the outcome of music intervention vary widely, and objective evidence of stress reduction has been lacking. Tible et al.^8^ emphasize that it remains unclear if non-pharmacological interventions are efficacious due to a shared activity such as positive human interaction. Improved human interaction is one of the goals in music interventions.

Stress hormone assessment has the potential to provide objective evidence of stress levels and thus establish the efficacy of music and other behavioral interventions.

### Stress endocrinology

Cortisol and DHEA-s are secreted in response to stress. Both are produced in the adrenal glands. Cortisol is the body’s primary stress hormone. DHEA-s, a conjugated steroid, is the sulfated metabolite of dehydroepiandrosterone. DHEA-s is secreted together with cortisol in energy-demanding situations.

Empirical evidence shows that chronic stress can cause physical changes in the brain that include atrophy of neurons near the hippocampus (the area in which memory and learning are centered) and expansion of neurons near the amygdala, which is responsible for threat awareness.^9^ Repeated assessments of the concentration of cortisol can be used as an index of adverse stress reactions in observational studies and in stress experiments has been documented in several studies.^10^ Assessments of saliva cortisol have also been used specifically in studies of stressful conditions in family members of PWD.^11^

DHEA-s may play a protective role during stress, as an antagonist to the neurotoxic consequences of elevated cortisol.^12^ However, with chronic stress the capacity to produce DHEA-s is reduced^13^ and DHEA-s levels are lower.^14,15^

The ratio of cortisol to DHEA-s (or DHEA) represents the balance between catabolic and anabolic activity and is an indicator of adverse chronic stress. A high cortisol/DHEA ratio is related to both chronic stress in caregivers^16^ and cognitive disorders.^17,18^ Its importance has been discussed in relation to cognitive decline due to chronic stress in caregivers of Alzheimer disease patients.^19^

In an effort to develop an objective measure of whether or not music reduces stress levels for either PWDs or their caregivers, we obtained saliva samples that enabled us to ascertain levels of cortisol and DHEA-s. The overall aim of the study was to measure stress reduction in PWDs and their FCGs through deployment a home-based music intervention. Reduction of endocrine stress levels should result with a decreased concentration of cortisol particularly in the evening and also with an improved ratio between energy mobilization (cortisol) and regeneration (DHEA-s). Evening cortisol is regarded as a useful indicator of accumulated stress reactions during the day.^20^ In subjects who are exhausted by long-lasting exposure to stress and have developed “flat curves” with small variations in cortisol over the day, there may be relatively low morning and high evening levels. Subjects who are exposed to high stress levels but are able to respond to this with marked elevation in cortisol excretion are likely to have high cortisol levels both in the morning and the evening. Change in evening cortisol was therefore regarded as the primary outcome in the present study whereas change in morning cortisol was more difficult to formulate a hypothesis about since some of the participants could have been living in a markedly stressful situation due to the cognitive illness for a long time before the study started.

Plasma concentration of cortisol and DHEA-s is mirrored in saliva.^21^ Since the collection of saliva is much less intrusive than the collection of blood samples, saliva sampling enables researchers to monitor a large number of samples that mirror both circadian and long-term variations in the concentration of these stress-related hormones.^10^

Morning saliva samples were also collected at awakening. They were used for a parallel analysis of cortisol, although the a priori interpretation of morning cortisol levels is more difficult than the interpretation of evening cortisol (see above). The cortisol awakening response, which requires a second morning sample, was not tested. However, the morning saliva sample at awakening was also used for analysis of DHEA-s, and diurnal cortisol variation was also analyzed (morning minus evening cortisol).

For circadian rhythm (difference between morning and evening cortisol) no a priori hypothesis could be formulated.^10,22^ A long-lasting chronic stress situation, with long-lasting sleep disturbance^23^ can result in physiological exhaustion corresponding to a flattened cortisol rhythm (small morning-evening difference). Both PWDs and FCGs can exhibit such patterns. In such a case, an improved psychosocial situation would result in an amplified difference due to a restored capacity to regulate stress hormones when psychosocial conditions improve. On the other hand, if the diagnosis of dementia in the patient is relatively recent, both PWDs and FCGs who have normal stress regulation capacity may be acutely stressed.

## Methods

### Participant recruitment and enrollment

An occupational therapist recruited 32 PWDs and their 32 FCGs via a memory evaluation center with a socioeconomically diverse population. The following inclusion criteria were used to select PWD participants: diagnosis of dementia made by a physician, a score of ≥4 on the Global Deterioration Scale, a score of ≥15 on the Brief Agitation Rating Scale, ability to hear normal conversation within two feet, and living with a family caregiver who could complete and/or provide assistance with saliva testing and implementation of the home-based music intervention (HBMI). PWDs were excluded if there were signs of active, untreated pain, infection, or other known medical condition that might cause neuropsychiatric symptoms.

Interested participants were first screened at the memory clinic or by phone. If qualified, participants were scheduled for an in-person (home) interview. On the assessment day, after signing the consent, the PWDs and FCGs were asked about demographic characteristics, use of psychotropic medications, and use of caregiver supportive services.

### Intervention

All selected PWDs and their FCGs were taking part in a music intervention program developed by FOU Nordost in collaboration with the Swedish Dementia Centre [http://www.demenscentrum.se/musik]. The purpose of the online program preparation is to explain the rationale for using music and provide the specific steps needed to determine the PWD’s musical preferences and the best ways to introduce and use music during personal care activities or other times throughout the day. The home-based program introduction takes about 2 h to complete, and the program can be accessed whenever convenient for the participants. The participants completed this introductory program before the actual musical intervention lasting for 2 months started. When the intervention period started the participants had decided, after participation in the introduction, their every-day listening schedule (for instance fixed hour every day or always after breakfast and dinner and always PWD and FCG together). All songs in the program are recorded in two versions, one with vocals and one as an instrumental version that can be used as accompaniment for singing. No prior knowledge of music is needed. During the intervention period the dyads mostly used the recorded pieces in the program but they were also free to use other recorded music during the scheduled daily music events.

### Saliva collection

Saliva collection is non-invasive and requires minimum equipment or expertise to perform. This makes it possible to collect multiple, sequential specimens from the same subject. Since the awakening concentration of cortisol in a subject with normal cortisol regulation capacity provides information about the amount of expected stress during the day, a proxy measure of this awakening response is the difference between morning and evening cortisol.^10^

Prior to starting the saliva collection, each family received individual or group education including a hands-on demonstration of proper techniques well as written, illustrated instructions for all steps, including the saliva collection, packing the saliva in plastic bags, and storing them in the home refrigerator until the next day’s pickup by a certified transportation company.

Participants collected saliva 5 days per week, from Sunday evening to Friday morning for eight “working” weeks started the same week as the music intervention began and ending when that intervention ended.

Participants were instructed to take the evening saliva sample before bedtime and before brushing their teeth and the morning saliva sample immediately after awakening. Timing of the morning sample was crucial because of the cortisol awakening phenomenon (CAR), in which cortisol level can rise as much as 75% in the first 30–45 min after awakening.^24^

The couples were recruited on an ongoing basis over the study period, which lasted from November 2019 to July 2019.

### Saliva transportation and analysis

Five days a week, the samples were picked up at the participants’ homes and transported to a biobank at the Karolinska Institute for pre-analytical storage until analyzed.

Cortisol and DHEA-s were analyzed by Truly Labs, a CRO (Contract Research Organization) laboratory specialized in biomarker analysis, using radioimmuno-assay.^25^ Commercial kits were from Salimetrics, State College, 16803 PA, USA. The intra- and inter-assay precision coefficients in the Truly lab. were 3.3% and 6.1% for cortisol and 3.1% and 2.3% for DHEA-s. The lowest detectable concentrations were 0.007 µg/dL (cortisol) and 43 pg/ml (DHEA-s) respectively.

### Statistical methods

At first, all participants were treated statistically as 47 (23 PWD and 24 FCG) independent samples of observations, with 15–40 observations for each individual. Thirteen of the PWD had 35–40 usable saliva samples; five had 25–34 usable samples, and five had 15–24 valid saliva samples. Twelve of the FCG had 35–40 usable samples, eight had 25–34, and four had 15–24 eligible samples. In one PWD the number of observation weeks was 6 (which was the lower limit we defined as sufficient) but the number of eligible saliva samples was too small (three) for the regression analyses, so this participant was excluded. The corresponding caregiver was included.

For each participant a linear regression coefficient was adjusted with four different outcomes versus time in days, namely 10 log [morning saliva cortisol] and 10 log [evening saliva cortisol], ratio 10 log  [morning cortisol]/10 log [DHEA-s] and ratio 10 log  [morning cortisol] minus 10 log  [evening cortisol]. The decision was made to define an individual’s regression as “improved over time” whenever a *t*-test indicated that the regression coefficient deviated “significantly from zero” in the “improving direction” (decreasing trend) using a one-sided test with *p* < 0.05. Similar one-sided *t*-tests were conducted to make a decision that the regression coefficient deviated “significantly from zero” in the “deteriorating direction” (increasing trend). This procedure was used for all outcomes except for the morning-evening difference for which a two-sided test was used. The reason why a two-tailed test was used for the morning-evening difference was that for this variable it was impossible to formulate a directional hypothesis.

In the next step the individual regression coefficients were used to test the mean linear trend by time on a group level using the one-sample *t*-test for the mean regression coefficient. A directional hypothesis (one-sided) was used for the primary outcome evening cortisol as well as for morning cortisol and cortisol-DHEA-s relationship trends. For the morning-evening trend a two-sided test was used, in consistency with the individual tests (see above). The type-I error rate of 0.05 was used, that is, concluding statistical significance if *p* < 0.05.

For all the four outcome variables a product-moment correlation coefficient was calculated between the PWD and the FCG regression coefficients regarding linear trend over time. We did this analysis in order to explore the extent to which the development over time (linear regression) in the PWD mimicked the development in the other member of the dyad, the FCG. The significance test for these correlations was two-sided with *p*-value ≤0.05.

Ethical approval was obtained from the Karolinska Institute’s institutional review board, Dnr: 2018/1596–31/2. Verbal consent was obtained from all participants.

## Results

Out of the 32 PWD/FCG dyads enrolled in the study, 23 PWDs (72%) and 24 FCGs (75%) completed the intervention for at least 6 weeks and were included in the analysis. Among the FCGs, 67% (n = 16) were female. Their ages varied from 37 to 90 years (mean 73, SD 11.28). Among the PWDs, 74% were male. Their age varied from 60 to 93 years (mean 78, SD 8.38). Months since being diagnosed with dementia varied from 5 to 57 with an average of 20 months (SD 13.72).

Table 1 illustrates the results of the individual regression analyses for the 23 PWDs and 24 FCGs. Our main hypothesis was that evening cortisol would show a decreasing (improving) trend during the intervention period. Using one-sided tests for individual evening cortisol, six (26%) of the PWDs and seven of the FCGs (29%) showed significantly improving levels; increasing (deteriorating) levels were found in two (4%) of the PWDs and three (13%) of the FCGs. The corresponding analyses for morning cortisol showed that five (22%) of the PWDs and six (24%) of the FCGs had a significantly improving coefficient, whereas significant positive (deteriorating) coefficients were found for morning cortisol among 2 (9%) of the PWDs and 1 (4%) of the FCGs.

**Table 1.** Summary of individual estimates of linear regression coefficients in 23 PWD and 24 FCG, for log10-cortisol by time, using one-sided test *p* ≤ 0.05.

	Cortisol			
	Persons with dementia		Family caregivers	
	Morning *n* = 23	Evening *n* = 23	Morning *n* = 24	Evening *n* = 24
	Mean (SD)	Mean (SD)	Mean (SD)	Mean (SD)
	−0.00039 (0.00261)	−0.00012 (0.00596)	−0.00087 (0.00583)	−0.00038 (0.00875)
	*n* (%)	*n* (%)	*n* (%)	*n* (%)
Individually significant negative regression coefficients*	5 (22)	6 (26)	6 (24)	7 (29)
Statistically significant positive regression coefficients**	2 (9)	2 (9)	1 (4)	3 (13)
Statistically non- significant regression coefficients	16 (69)	15 (65)	17 (71)	14 (58)

SD: standard deviation.

* An individually significant negative regression coefficient corresponds to a successive (expected) reduction in cortisol level during the intervention period.

** An individually significant positive coefficient indicates that the linear trend is in the opposite (unexpected) direction.

**Figure 1.**
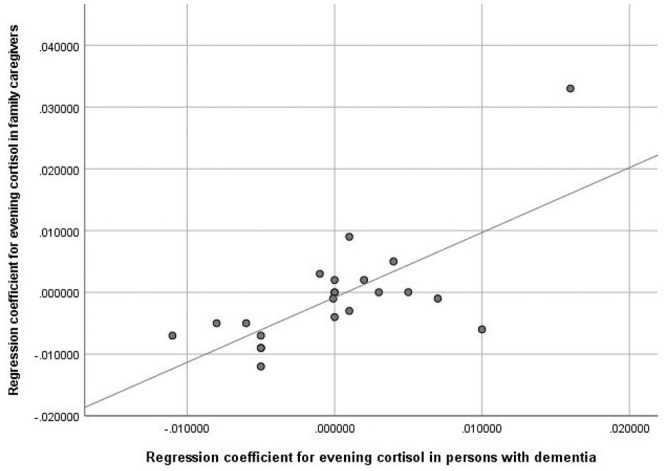
Correlation between person with dementia (x) and family caregiver (y) trend (individual regression over time) with regard to evening cortisol development. *r* = 0.71, *p* < 0.001, two-sided.

The correlation coefficient of PWDs and FCGs for the regression coefficient regarding the linear trend over time for evening cortisol was *r* = 0.71 (*p* = 0.001, two-tailed) (Figure 1). The corresponding dyad correlations for morning cortisol as well as for the cortisol/DHEA-s ratio were not significant.

The four mean regression coefficients (morning PWD, evening PWD, morning FCG and evening FCG curves) on a group level did not show any significant trends over time, according to one-sided *t*-tests for the mean linear regression coefficient (morning PWD: *t* = −0.736, *df* = 22, *p* = 0.23, evening PWD: *t* = −0.211, *df* = 22, *p* = 0.42, morning FCG: *t* = −0.720, *df* = 23, *p* = 0.24, evening FCG: *t* = 0.102, *df* = 23, *p* = 0.46).

Table 2 illustrates the ratio between log cortisol and log DHEA-s. In 22% of the PWD and 25% of the FCG the results show negative trends (improvement). An improvement of the cortisol/DHEA-s ratio corresponds to improved balance between energy mobilization and regeneration. There was no significant trend on the overall group level according to a one-sided *t*-test (log cortisol – log DHEA PWD: *t* = −0.767, *df* = 22, *p* = 0.23, log cortisol – log DHEA FCG: *t* = 0.957, *df* = 23, *p* = 0.17).

**Table 2.** Summary of individual ratios of individual estimates for linear regression coefficients 10 log [cortisol]/10 log DHEA-S. One-sided *p* ≤ 0.05.

	Persons with dementia	Family caregivers
	Ratio 10 log [cortisol]/10 log[DHEA-S] *n* = 23	Ratio 10 log [cortisol]/10 log[DHEA-S] *n* = 24
	Mean (SD)	Mean (SD)
	−0.00017 (0.00111)	0.00147 (0.00740)
	*n* (%)	*n* (%)
Individually significant negative regression coefficients*	5 (22)	6 (25)
Individually significant positive regression coefficients**	2 (9)	3 (13)
Individually non-significant regression coefficients	16 (69)	15 (62)

* An individually negative regression coefficient corresponds to a successive (expected) reduction in cortisol level during the intervention period.

** An individually positive coefficient indicates that the trend is in the opposite (unexpected) direction.

Table 3 presents individual regression coefficients for both PWDs and FCGs for circadian rhythm. The individual regression coefficients did not show any particular patterns for cortisol “slope” (log cortisol morning -log cortisol evening difference). However, the analysis of group trends for these coefficients with the use of two-sided *t*-tests shows that among FCGs there is a statistically significant trend toward a diminished cortisol slope during the study period (*t* = −2.260, *df* = 23, *p* = 0.03). However, there is no corresponding significant group trend in the PWD group (*t* = −0.347, *df* = 22, *p* = 0.73).

**Table 3.** Circadian rhythm (log cortisol morning minus log cortisol evening) two-sided *p* < 0.05.

	Persons with dementia	Family caregivers
	Circadian rhythm*n = 23	Circadian rhythm*n = 24
	Mean (SD)	Mean (SD)
	−0.00060 (0.00842)	−0.00733 (0.01590)
	*n* (%)	*n* (%)
Individually significant negative regression coefficients**	1 (4)	2 (8)
Individually significant positive regression coefficients***	1 (4)	1 (4)
Individually non-significant regression coefficients	21 (91)	21 (88)

* Log cortisol morning minus log cortisol evening.

** An individually negative regression coefficient corresponds to a successive reduction in cortisol level during the intervention period.

*** An individually positive coefficient indicates that the trend is in the opposite direction.

Readers who wish to have more information regarding distribution of regression coefficients as well as intercepts and other statistical information are invited to contact the main author.

### Individual cases

In order to illustrate in a more concrete way how the regression coefficients were generated we present illustrative narrative descriptions of two dyads, with diagrams of the cortisol results. Note that the diagrams show the concentration in ng/l while all the statistics have been based upon 10 log transformation of these concentrations.

**Figure 2.**
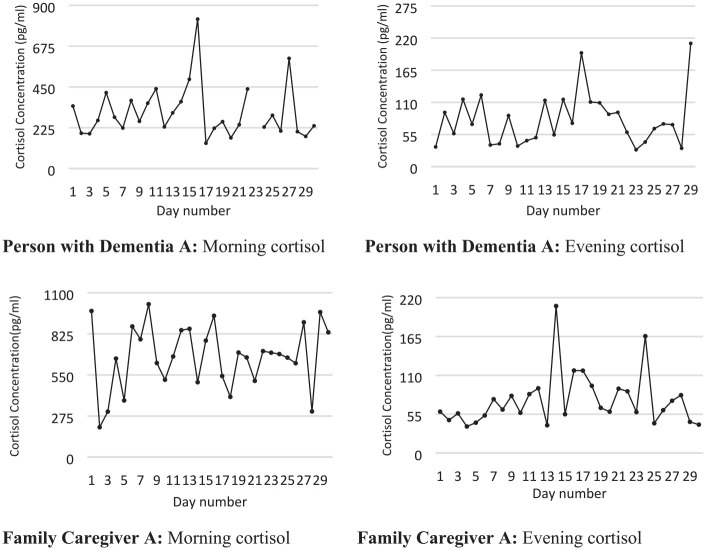
Saliva cortisol morning and evening concentration in one dyad of person with dementia and family caregiver.

Figure 2 illustrates PWD A and FCG A morning and evening cortisol levels during 29 saliva collection days. The PWD was a man in his early 80s. He had been an entrepreneur with a successful business and retired from his business a couple of years prior to the study period. He lived with his same-aged wife. His wife had noticed a gradual deterioration in the PWD’s condition and behavior during the past 10 years. The diagnosis of Alzheimer’s disease was made 5 months before the intervention started. At the study enrollment, he was rated as having moderately severe cognitive decline (GDS = 5) by the research assistant. The PWD objected to the diagnosis, stating “I have no dementia.” After his retirement from business, he was passive and uninterested in all kinds of activities. The PWD showed deteriorating gait. There was a history of alcohol and medication abuse. The wife took responsibility for limiting his abuse.

The amount of music listening was slightly irregular during the first half of the intervention period but became more regular during the second half and occurred twice during the day and once in the evening.

The PWD preferred to listen to classical music, while his wife liked to listen to pop music. The wife worried because the PWD lacked motivation to listen and three extra home visits were made by the project’s occupational therapist. At one of these visits the PWD was violent, threw things around and shouted. He calmed down when listening to classical music. He had an occasional hot temper, which had worsened.

In the middle of the intervention period, the PWD had an infection with high fever that amplified his violent behavior. During the intervention there was an improvement in the PWD’s behavior. He accepted his diagnosis and stated that he was satisfied. Bouts of aggression decreased as did suspiciousness directed toward his wife.

There were no systematic changes in the cortisol curves of either the PWD or the caregiver. In the PWD we can see bursts of cortisol activity and an overall unstable picture.

**Figure 3.**
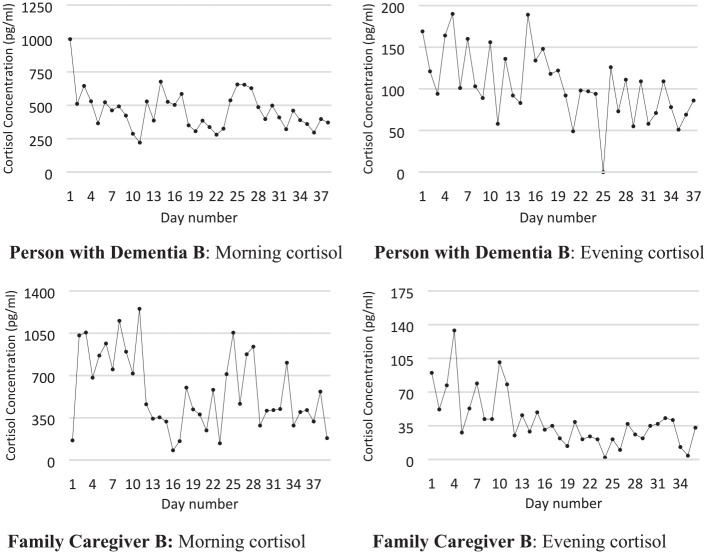
Saliva cortisol morning and evening concentration in another dyad of person with dementia and family caregiver.

Figure 3 illustrates morning and evening cortisol levels for PWD B and FCG B during 37 saliva collection days. The PWD is a woman in her early 70s. She lives with her husband, who is in his mid-70s. She was diagnosed with frontal lobe dementia 30 months before the start of the intervention. At study enrollment she was rated as having severe cognitive decline (GDS = 6) by the research assistant. Both executive function and short-term memory were affected at the time of the intervention. She did not have insight. She claimed that she was not ill at all. This caused conflicts at home. She could not manage walks on her own due to problems with orientation and because of knee arthrosis. She had pronounced suspiciousness about the environment and was easily angered. The PWD also had difficulties in staying focused.

The pair chose to listen to music more or less continuously during the day and to use special music at meals. They chose dance music and several ABBA pieces and picked up several of their own CDs for listening.

During the intervention the PWD started gardening with her husband, so she was spending much more time outdoors. She joyfully welcomed visitors. Her husband also showed improved mood.

The PWD’s cortisol concentration shows a significant decreasing (improving) trend both in the evening and the morning. The husband’s cortisol concentration also shows a significant downward (improving) trend both in the morning and in the evening.

The ratio between cortisol and DHEA-s did not change significantly in the PWD but did change significantly in the improving direction for the FCG.

### Comments on descriptions of the two individual dyads

The first dyad illustrates that the intervention period was problematic for many reasons, both for the PWD and the FCG. An infection with high fever in the PWD disturbed both the intervention and data collection. The diagnosis was fairly recent. The diagnosis may in itself have resulted in adaptation to a new situation, although they had been living with increasing difficulties for a long time. The PWD was not very collaborative in music listening. No significant endocrine changes were observed.

The second dyad was quite different. In this case the diagnosis had been known for a much longer time – 30 months. The intervention was successful and the music listening had a pronounced effect on the relationship, which improved considerably. In this case significant beneficial endocrine changes were observed in both the PWD and FCG.

## Discussion

The results show that about one-fourth of the PWDs and one-fourth of the FCGs had a significant downward trend (improvement) in evening cortisol levels. A reasonable interpretation is that these subjects experienced decreasing stress levels during the intervention period. A similar proportion in both groups also experienced a decreasing (improving) morning cortisol during this period. On a group level the morning-evening differences decreased significantly in the FCG group. These two latter observations speak against the possibility that any substantial part of the participants had been exhausted, with “flat cortisol curves” initially. This is particularly the case for the FCG group, but since the dominant pattern is decreasing cortisol levels both in the evening and morning, an “exhausted” start is unlikely. Instead, it is likely that an initially high stress level elicited increased cortisol excretion throughout the day in individuals with retained capacity to respond to stress. In general, the results are more consistent for FCGs than for PWDs, which could be due to deterioration in the PWDs’ illness during the study period as illustrated in the case histories. In each of the dyadic groups, one to three participants had a significant development of cortisol concentration in an unexpected direction either in the morning or evening, and in more than half of the participants the direction could not be clearly determined.

The analyses of the ratio between cortisol and DHEA-s showed that five PWDs and six FCGs had a statistically significant successive improvement of the balance between energy mobilization and regeneration during the study period, but two PWDs and three FCGs showed a development in the opposite direction.

The analyses of circadian cortisol rhythm showed that while there was no systematic change at all in the PWDs, there was an overall significant decrease over time for the FCG group in cortisol slope, possibly indicating that FCGs had progressively decreasing physiological stress level during the study period. This provides additional support, at least in the FCG group for the notion that the “exhausted” pattern was not prevalent initially.

The timing of the “upon awakening” sample is subject to variation. Delays could have occurred and some subjects may have taken time before they got out of bed after they woke up. However, this definition has become standard in the literature.^10^

Our decision was to not analyze the cortisol awakening response because of the challenges with data collection in this study. However, it should be pointed out that the morning-evening cortisol slope is correlated with the awakening response.^26^ Thus, since there was a decreasing amplitude in the morning-evening difference during the intervention period we might have found a decreasing awakening response as well.

With the exception of one of the caregivers, a 37-year old man, both PWDs and FCGs in both groups were old (from 60 to 90). Because of the high mean age, neither age nor gender had a statistically significant relationship with cortisol or DHEA-s concentration. However, the 37-year old man had a much higher DHEA-s concentration than the other participants, as expected. Any influence of the higher mean levels in this subject was minimized by the logarithmic transformation of hormone concentrations in all the statistical analyses.

To date, a control group (without intervention) has not been compared with the intervention group. We thus do not know whether or not the physiological signs of decreased stress observed in some dyads were due to the music intervention program. A more detailed analysis of factors of importance for physiological improvement will be published at a later time. The experiences self-reported by caregivers illustrate that in the successful dyads the relationship between the PWD and FCG improved as a likely consequence of the music listening. The introduction was tailored to each dyad’s needs.

Improvement of stressful conditions cannot be expected with no intervention in a sample of PWDs with moderate dementia. This was confirmed in a study^11^ in which an intervention inspired by cognitive behavioral therapy was tested for FCGs of PWDs. Saliva cortisol concentration was assessed in these caregivers on four occasions during the day (from awakening until bedtime) during two assessment days – before the intervention course and 2 months later. In the control group there was no change in cortisol levels during the intervention period, while significantly decreased levels were observed in the intervention group.

The benefit of music intervention in institutionalized care has been reported,^5–7^ and there is no reason to believe that it would not play an equal or even greater role in home care.

That the FCG group showed decreasing difference between morning and evening levels of cortisol could mean that this group started with a healthy stress response potential and a high stress level, which declined. This pattern was not seen in the PWD group. Why there is such a difference between PWDs and FCGs is not known. One reason could be that the FCGs were better able to regulate cortisol excretion than the PWDs.

The fact that evening cortisol and not morning cortisol showed parallel trends in PWDs and FCGs might be because morning cortisol levels are more influenced by genetic factors than are evening levels.^22^

The decreases in morning levels observed in the significantly “de-stressed” (improved) cases (defined as significantly decreased cortisol) were on the order of 100–400 pg/ml, which is a substantial change. Figures 2 and 3 show that cortisol levels fluctuate considerably and hence many assessments are necessary – although the number of samples collected in our example may be unnecessarily high. As illustrated in the study, the collection of saliva samples is easy to perform and if collaboration with the caretaker is successful the patient is able to cooperate in the sampling procedures. Thus, 72% of the initially recruited dyads completed the data collection according to the plan (75% of the FCGs).

### Strengths

Acceptance of the saliva measurement procedure was high. Despite the demands of collecting twice a day for 2 months, about three-fourths of the participants collected a satisfactory number of samples. This indicates high acceptability.

The number of observations was large for each participant. This made it possible for us to observe variability patterns and test trends during the intervention period. Each individual’s data could be regarded as a separate experiment and hence 47 experiments and statistical tests were completed for each outcome.

We observed both PWDs and FCGs. A general observation was that the intervention seemed to be slightly more beneficial for the FCGs than for the PWDs. The trends in evening cortisol showed dyadic correspondence.

Cortisol concentration was examined both in the morning and in the evening. This made it possible to identify circadian patterns–a significant decrease in cortisol morning-evening slope was observed on a group level in the caregivers. In individual cases it was also possible to analyze specific morning and evening problems respectively. Our data indicate some individuals had a decrease in morning cortisol only, while others had a decrease in only their evening cortisol level. A detailed analysis will be presented in a coming publication.

A final strength was that we included DHEA-s in the analyses. This made it possible to test whether the balance between energy mobilization and regeneration improved.

### Limitations

The two major limitations are that we had no control group and the number of study participants in both groups was relatively small. It was impossible to perform a power calculation because there were no previous publications indicating expected effects with this intervention and these measurements.

As indicated from other literature, it is unlikely that PWDs and FGCs not participating in any particular intervention program would have shown improvements as measured by these biomarkers.

## Conclusions

Morning and evening cortisol and DHEA-s measurements can provide an objective measure of stress levels and have an important role to play in assessing the impact of a behavioral intervention such as music on the stress being experienced by PWDs and their caregivers. This methodology is also applicable to other kinds of studies involving behavioral interventions.

This was a pilot study that confirmed the ability of non-professionals to reliably collect, store, and transmit saliva samples that yielded data on cortisol and DHEA-s levels. Accomplishing this relied on a carefully constructed plan of PWD and caregiver education, and the continuous availability of a professional for consultation when questions arose on the part of the caregiver responsible for sample collection.

Reductions in stress, as measured by cortisol and DHEA-s, were observed in one-fourth of the participants. In the FCG group there was a significant decrease in the cortisol morning-evening difference. Although no causal inference can be made in this study regarding the effect of the music intervention, our findings show that this method for recording endocrine stress is helpful for the unbiased assessment of a behavioral intervention.

Interpretation of the data and application of the methodology require continued development and validation.
